# No increased risk of spinal cerebrospinal fluid leak after spinal manipulative therapy: A retrospective cohort study

**DOI:** 10.1002/pmrj.70072

**Published:** 2025-12-26

**Authors:** Robert J. Trager, Collin M. Labak, Anthony N. Baumann, Tyler H. Snodgrass, Zachary A. Cupler

**Affiliations:** ^1^ Connor Whole Health University Hospitals Cleveland Medical Center Cleveland Ohio USA; ^2^ Department of Family Medicine and Community Health, School of Medicine Case Western Reserve University Cleveland Ohio USA; ^3^ Department of Biostatistics and Bioinformatics Clinical Research Training Program Duke University School of Medicine Durham North Carolina USA; ^4^ Neurosurgery University Hospitals Cleveland Medical Center Cleveland Ohio USA; ^5^ Department of Rehabilitation University Hospitals Cleveland Medical Center Cleveland Ohio USA; ^6^ College of Medicine Northeast Ohio Medical University Rootstown Ohio USA; ^7^ Department of Orthopaedic Surgery, Cleveland Clinic Akron General Akron Ohio USA; ^8^ Physical Medicine and Rehabilitation Services VA Finger Lakes Healthcare System Rochester NY USA; ^9^ Physical Medicine and Rehabilitation Services, Butler VA Health Care System Butler Pennsylvania USA

## Abstract

**Background:**

Spinal cerebrospinal fluid (CSF) leaks, a rare but debilitating condition, have been described following spinal manipulative therapy (SMT) in case reports. However, the nature of the potential association between SMT and CSF leak is uncertain, and symptoms such as neck pain or headache may reflect preexisting leaks rather than effects of SMT.

**Objective:**

To test the hypothesis of no significant association between SMT and spinal CSF leak, comparing chiropractic SMT recipients to matched controls undergoing physical therapist‐supervised therapeutic exercises (PTE).

**Methods:**

We searched a health‐records‐based U.S. dataset (TriNetX, LLC) for adults with spinal disorders receiving an index event of (1) SMT or (2) PTE without SMT from 2005 to 2024, excluding those with preexisting CSF leaks and related conditions, using propensity matching to minimize confounding. Our primary outcome was the risk ratio (RR) of spinal CSF leak occurring within 2‐month follow‐up. A secondary composite outcome included the diagnosis of spontaneous intracranial hypotension in addition to the primary outcome.

**Results:**

After matching, 89,042 patients remained per cohort. There was no significant association between SMT and spinal CSF leak incidence or risk compared to PTE (both cohorts: 0.03% [95% confidence interval: 0.02%–0.04%]; RR = 0.85 [0.49–1.49]; *p* = .572). Adding spontaneous intracranial hypotension to the primary outcome yielded similar results.

**Conclusions:**

The present findings suggest chiropractor‐administered SMT does not increase the risk of spinal CSF leak in adults with spinal disorders relative to PTE. Although our analysis focused on chiropractor‐delivered SMT, the findings could plausibly apply to SMT delivered by other clinician types, warranting further research. Given the overlapping symptoms of CSF leak and musculoskeletal neck pain or headache, clinicians who manage spinal disorders should be aware of this condition.

## INTRODUCTION

Spinal cerebrospinal fluid (CSF) leaks are a debilitating condition characterized by CSF egress through a defect in the dura mater, the protective outer layer of the central nervous system, which can lead to spontaneous intracranial hypotension (SIH).^1^ Although rare, with an estimated incidence of 5 per 100,000 person‐years,^2^ SIH can cause significant morbidity and may require interventional repair via blood patch or primary surgical repair in refractory cases. Possible sequelae of SIH include orthostatic headaches, and if left untreated, intracranial hemorrhage, which can cause profound neurologic deficit.^2^, ^3^ Spontaneous spinal CSF leaks typically affect middle‐aged adults, having a mean age of 52.4 years, and most commonly involve the thoracic spine.^4^

Several case reports have raised questions about a potential association between spinal manipulative therapy (SMT), a hands‐on treatment often used by chiropractors for spinal pain,^5^ and spinal CSF leaks. A 2022 review identified 13 cases of spinal CSF leak and/or SIH following SMT, with patients having a mean age of 41.6 years (SD = 7.8 years) and female predominance (83.3%).^6^ Among the five cases where CSF leak location was identified, all occurred in the thoracic (*n* = 3) and cervical (*n* = 2) regions.^6^ Some authors have hypothesized that the mechanical forces of SMT might lead to dural tears in the presence of spinal osteophytes (ie, bone spurs)^7^ or strain the ligamentous tissue covering the nerve root(s).^8^ However, these proposed mechanisms remain speculative in light of the reliance on sporadic case reports, which have an inherent limited ability to evaluate meaningful correlation.^9^, ^10^, ^11^

The symptoms of spinal CSF leaks and SIH substantially overlap with those produced by common neuromusculoskeletal conditions. Among patients with SIH, 97% report headache, 43% have neck pain or stiffness, and 14% report back pain.^3^ These symptoms are also frequently evaluated and treated by chiropractors.^12^ This symptom overlap raises the potential of protopathic bias, where early‐stage spinal CSF leak and SIH symptoms may have prompted the patient to seek care, rather than SMT being causative of the CSF leak.^9^ Illustrative case reports have described patients with undiagnosed SIH presenting to the chiropractic office.^13^, ^14^ Given the challenges in deriving conclusions from case reports alone and examining a relatively rare condition, adequately powered studies with methods to minimize observational biases are needed to examine this subject.

To date, no large‐scale studies have examined the potential association between SMT and spinal CSF leaks. Given the clinical relevance of understanding potential risks associated with SMT, further investigation was warranted. We aimed to address this gap by conducting a matched retrospective cohort study comparing the risk of developing a spinal CSF leak between adults receiving chiropractic SMT and those undergoing physical therapist‐supervised therapeutic exercise (PTE) without SMT. Given the limited evidence on the topic, we examined the association between SMT and spinal CSF leak, testing the null hypothesis of no significant association.

## MATERIALS AND METHODS

### Study design

This retrospective cohort study included patients meeting selection criteria from December 20, 2005 through December 20, 2024, with a query date of February 20, 2025, to allow for 2 months follow‐up. We used the TriNetX network, a federated platform that provides deidentified, aggregate‐level data from large health care organizations in the United States (TriNetX, LLC., Cambridge, MA, USA). We adhered to a registered protocol.^15^

The network is chiefly based on electronic health records and is compliant with privacy and security regulations, including the Health Insurance Portability and Accountability Act. TriNetX allows for retrospective research of routinely collected data, enabling queries across standardized nomenclatures like the *International Classification of Diseases, Tenth Revision* (ICD‐10) and Current Procedural Terminology (CPT). This platform includes over 142 million patients, and both insured and uninsured individuals from 105 health care organizations. The first author's institutional review board (Cleveland, OH, USA) considers studies using de‐identified data from the online TriNetX platform ‘Not Human Subjects Research’ thereby exempting the present study from institutional review board review and waiving the need for consent.

### Patient sample

#### Inclusion criteria

We included adults (age ≥ 18 years) with a range of spinal disorders (ICD‐10: M50‐M54 [other dorsopathies]), including those with neck pain, mid‐back pain, low back pain, and/or spinal disc disorders, which are common conditions that patients present with to both chiropractors and physical therapists.^12^, ^16^, ^17^, ^18^

Patients were divided into two cohorts according to the first co‐occurrence (index visit) of either a spinal disorder and (1) chiropractor‐administered SMT (CPTs: 98940, 98941, 98942) or (2) PTE (CPTs: 1029677 or 97001 [physical therapy evaluations] and 97110 [therapeutic exercise]). We also required that patients have a preceding health care visit of any kind between 1 week and 1 year prior to the index visit, to improve data completeness. Preceding health care visits were broadly defined and could include any encounter type recognized by TriNetX, such as ambulatory, emergency, inpatient, observation, laboratory, pharmacy, or virtual visits. This requirement was intended to ensure patients had engagement with the health care system and had data prior to the index date to allow application of eligibility criteria and propensity score matching.

The present study focused exclusively on SMT administered by chiropractors for several reasons. Chiropractor‐administered SMT has a specific set of CPT codes that are easily identified in the electronic health records,^19^ exclusive to treatment of the spine, and sufficiently represented in the TriNetX dataset with approximately 150,000 unique patients. Other codes that may be used to represent SMT administered by different clinician types, such as manual therapy (CPT: 97140) may also be appended for non‐SMT therapies, such as manual lymphatic drainage and manual traction.^20^ Osteopathic manipulative treatment codes, also represented by CPT codes in the TriNetX platform, are subdivided by body regions yet are not inherently spine specific and could be used in cases of treatment of viscera or the extremities.^21^ Accordingly, our use of chiropractic SMT codes was purely methodological given the coding specificity, sample size, and relative homogeneity of the procedure.

Our strategy of using PTE as an active comparator aimed to minimize bias^22^ by ensuring that both cohorts were similarly engaged in the health care system for care of spinal disorders. Generally, patients who visit physical therapists for spinal disorders more closely resemble those visiting chiropractors than those visiting medical physicians or other specialists regarding comorbidities, self‐rated health status, socioeconomic factors, and insurance coverage.^23^ Both chiropractors and physical therapists are portal‐of‐entry providers that commonly care for spinal disorders and offer nonpharmacological interventions.^17^, ^18^ Additionally, therapeutic exercises are commonly used by physical therapists and recommended for treatment of various spinal disorders by clinical practice guidelines.^24^, ^25^ Although sports activities or even gentle exercises like yoga have been suggested as occasional potential precipitating factors in spontaneous CSF leaks, exercise is generally not viewed as a major risk factor for this condition.^26^

#### Exclusion criteria

We excluded patients with previous CSF leak, SIH, dural tear, orthostatic headache, CSF shunt, and those with recent spinal puncture, spinal cord stimulator procedures, spinal surgery or injection to broadly avoid including patients with preexisting CSF leaks.^3^, ^27^, ^28^, ^29^ We also excluded those with serious spinal pathology such as spinal infection, malignancy, or fracture, to minimize selection bias related to differences in spinal disorder complexity between cohorts, considering SMT is a contraindication in these scenarios.^30^ We excluded patients who received SMT (delivered by chiropractors), manual therapy (delivered by any clinician), or osteopathic manipulative therapy from the PTE cohort to minimize exposure misclassification. Therapeutic exercise (CPT 97110) and other physical therapy‐related procedural codes were not excluded from the SMT cohort because (1) provider type is often not specified in TriNetX and (2) chiropractic SMT is often delivered alongside exercise in real‐world care.^12^ Eligibility criteria are summarized in Supplementary File S1, Figure S1 and Table S1.

### Variables

#### Potential confounders

To minimize bias,^31^ we propensity matched cohorts based on key confounding variables that have a known association with spinal CSF leak and/or SIH, such as overweight and obesity, Ehlers–Danlos syndrome, and others (Supplementary File S1, Table S2). Variables present within 1 year preceding the index date were eligible for matching. Although more recent surgeries were excluded, we matched on further remote surgeries occurring over 2 months prior to the index date, considering the possibility of delayed spinal CSF leak and/or SIH after surgery.^32^ To account for potential between‐cohort differences in access to or use of diagnostic imaging, we controlled for socioeconomic markers and use of magnetic resonance imaging of the brain and spine and contrast agents, and claustrophobia.^2^, ^33^ We also matched on emergency, observation, and inpatient visits to further minimize potential differences in health complexity and health care use.^34^ To minimize potential time‐related biases in relation to possible advancements in diagnosis of spinal CSF leak, we matched cohorts based on their age at index and age at the time of query. This helped ensure a similar data range between cohorts.

#### Primary outcome

Our primary outcome was the occurrence of spinal CSF leak, defined as the occurrence of any of a composite of diagnoses and treatments related to spinal CSF leaks and dural tears (Supplementary File S1, Table S3). This approach accounts for variability in treatment and coding and aims to allow for sufficient case identification to enable statistical comparison. We used a 2‐month outcome window to give time for identification of CSF leak, which is often delayed. Prior cases described diagnosis of spinal CSF leak and/or SIH up to 4–5 weeks following SMT,^35^, ^36^, ^37^, ^38^ and in general, the median time to SIH diagnosis is 5 weeks.^34^ We intentionally avoided diagnosis codes describing intracranial CSF leaks and iatrogenic etiologies of CSF leak such as spinal puncture, ventricular shunting, or accidental dural plane violation during surgical procedures, and puncture‐related leaks from spinal procedures or anesthesia during labor. This outcome also does not include orthostatic headaches, which may arise from other conditions.

#### Secondary outcomes

Secondarily, we explored the risk ratios (RR) and total and cumulative incidence of an expanded outcome that includes patients identified by the primary outcome codes, as well as those having a diagnosis of intracranial hypotension and/or intracranial pressure of less than or equal to 4 mm Hg (Supplementary File S1, Table S4).^29^ These outcomes are added to determine if they yield additional cases potentially related to occult CSF leaks.^2^ Added markers of intracranial hypotension therefore serve as a sensitivity analysis and are not relied upon for hypothesis testing due to their lack of reliability and specificity for diagnosing spinal CSF leak.^2^ Occasionally, low CSF pressure can stem from various other etiologies including cranial CSF leak, CSF hypovolemia, or rare etiologies like CSF‐venous fistula or inferior vena cava obstruction.^39^, ^40^, ^41^, ^42^ We also reported the mean and median number of follow‐up chiropractic SMT visits in the SMT cohort and mean and median number of follow‐up physical therapy visits in the PTE cohort for descriptive purposes, also comparing the likelihood of these physical therapy visits between cohorts (Supplementary File S1, Table S5).

### Statistical methods

We compared the baseline characteristics and conducted matching via the TriNetX online analytics platform, using a standardized mean differnce (SMD) threshold of >0.1 as indicative of meaningful between‐cohort imbalance.^43^ TriNetX conducts greedy nearest‐neighbor propensity score matching using a 1:1 ratio and a caliper of 0.1 pooled standard deviations of the logit of the propensity score. A logistic regression was applied using Python (scikit‐learn version 1.3 [Python Software Foundation, Delaware, USA]) to pooled covariate matrices to predict the probability of each patient being in the PTE cohort (ie, propensity score of 1). The matching process iterated through patients in the smaller cohort, finding the closest available matches from the larger cohort and trimming patients who remained unmatched. Because the TriNetX platform returns aggregate rather than patient‐level data, formal imputation and missingness modeling were not feasible. However, eligibility criteria were designed to maximize data availability.

We used R (build 4.3.2, Vienna, AT^44^) to calculate (1) RRs with 95% confidence intervals (CIs), (2) spinal CSF leak risk difference and its 95% CI using the Miettinen and Nurminen method via the DescTools package,^45^ and (3) *p* values for RRs using the *χ* ^2^ test with a significance threshold of .05. We used ggplot2^46^ to plot spinal CSF leak total and cumulative incidences by cohort.

TriNetX censors patients the day after their last recorded clinical fact if they contribute no further information within the analysis window. This censoring may affect the cumulative incidence curves derived from Kaplan–Meier data provided by TriNetX (e.g., with widening 95% CIs over time) but did not influence our primary methods of analyses, which were based on incidence proportions without adjustment for censoring.

### Data quality assessment

We provided markers of data quality and matching diagnostics including reporting measures of cohorts' follow‐up duration (mean [compared via SMD], median, and interquartile range), the proportion of unknown demographics, and plots of covariate balance and propensity score density using ggplot2.^46^

### Negative control outcomes

We also explored RRs for negative control outcomes including azithromycin prescription (RxNorm: 18631), colonoscopy (CPT: 1022231), TDAP vaccine (vaccine code CVX 115: tetanus, diphtheria, and pertussis), and diagnostic imaging (CPT: 1010251).^47^, ^48^ These were evaluated by assessing whether the majority of point RR estimates approximate the null (0.73 ≥ RR ≤1.38), suggestive of between‐cohort balance.^48^, ^49^ Negative control outcomes were selected which represent routine health care procedures and are unrelated to SMT or PTE.^48^ Additionally, a similar likelihood of diagnostic imaging would point against surveillance bias.

### Required study size

We calculated a required total sample size of 155,814 using a *z*‐testing function in G*Power (Kiel University, Germany) to examine a difference in incidence proportion between cohorts of 0.0005 (0.0005 vs. 0.0010) to power our primary outcome using a power of 0.95, allocation ratio of one, and a two‐tailed α‐error of .05. This calculation relies on a previous general epidemiologic estimate,^2^ considering there are no large studies specific to this association to our knowledge.

## RESULTS

### Patients

Before propensity matching, there were 89,753 patients in the SMT cohort and 1,527,758 in the PTE cohort. After matching there were 89,042 patients per cohort. Before matching, the PTE cohort had a greater mean age, a greater proportion of patients who had an inpatient or emergency visit, and greater proportion who had undergone diagnostic imaging or had been administered iodine‐based contrast agents, among other differences (SMD >0.1). After matching, all key variables were optimally balanced (SMD <0.1) (Table 1).

**TABLE 1:** Baseline characteristics before and after propensity score matching.

	Before matching			After matching		
Variable *n* (%) or mean (SD)	SMT	PTE	SMD	SMT	PTE	SMD
*N*	89,753	1,527,758	NA	89,042	89,042	NA
Age at index, y	49.4 (16.6)	58.2 (16.1)	0.543	49.5 (16.5)	49.2 (16.7)	0.020
Age at query date, y	58.5 (17.0)	63.6 (15.9)	0.307	58.6 (17.0)	58.1 (16.9)	0.026
Female	55,145 (61%)	927,427 (61%)	0.015	54,797 (62%)	54,829 (62%)	0.001
Male	34,606 (39%)	563,069 (37%)	0.035	34,243 (38%)	34,147 (38%)	0.002
Adverse socioeconomic and psychosocial factors	652 (1%)	41,095 (3%)	0.152	651 (1%)	600 (1%)	0.007
Ankylosing spondylitis	107 (0%)	3863 (0%)	0.031	107 (0%)	108 (0%)	0.000
Arnold‐Chiari syndrome	21 (0%)	672 (0%)	0.011	21 (0%)	27 (0%)	0.004
Bariatric surgery	41 (0%)	1006 (0%)	0.009	41 (0%)	29 (0%)	0.007
Benign intracranial hypertension	93 (0%)	2258 (0%)	0.012	93 (0%)	76 (0%)	0.006
Cervical disc disorders	3488 (4%)	73,912 (5%)	0.047	3472 (4%)	3028 (3%)	0.027
Cervicalgia	13,517 (15%)	198,480 (13%)	0.060	13,349 (15%)	12,787 (14%)	0.018
Claustrophobia	144 (0%)	6565 (0%)	0.050	144 (0%)	140 (0%)	0.001
Diagnostic imaging (spine/pelvis)	20,907 (23%)	510,048 (33%)	0.225	20,864 (23%)	19,354 (22%)	0.041
Disc disorder (Thoracic/lumbar/lumbosacral)	8162 (9%)	180,525 (12%)	0.089	8134 (9%)	7676 (9%)	0.018
Ehlers–Danlos syndromes	81 (0%)	2887 (0%)	0.026	81 (0%)	61 (0%)	0.008
Headache	7963 (9%)	138,828 (9%)	0.008	7894 (9%)	7274 (8%)	0.025
Gadolinium MRI contrast agent	71 (0%)	9064 (1%)	0.089	71 (0%)	81 (0%)	0.004
Hypermobility syndrome	65 (0%)	1818 (0%)	0.015	65 (0%)	65 (0%)	0.000
Iohexol	1058 (1%)	167,445 (11%)	0.419	1058 (1%)	1113 (1%)	0.006
Marfan syndrome	14 (0%)	427 (0%)	0.008	14 (0%)	20 (0%)	0.005
Migraine	6263 (7%)	99,893 (7%)	0.018	6226 (7%)	5744 (6%)	0.022
MRI brain without contrast	1078 (1%)	63,173 (4%)	0.183	1078 (1%)	937 (1%)	0.015
MRI cervical spine	1838 (2%)	54,271 (4%)	0.091	1838 (2%)	1591 (2%)	0.020
MRI lumbar spine	3546 (4%)	94,008 (6%)	0.101	3545 (4%)	3345 (4%)	0.012
MRI thoracic spine	549 (1%)	17,697 (1%)	0.058	549 (1%)	471 (1%)	0.012
Neurofibromatosis, type 1	10 (0%)	384 (0%)	0.010	10 (0%)	19 (0%)	0.008
Orthostatic hypotension	369 (0%)	24,845 (2%)	0.121	368 (0%)	334 (0%)	0.006
Other central nervous system disorders	53 (0%)	2951 (0%)	0.038	53 (0%)	64 (0%)	0.005
Overweight and obesity	13,761 (15%)	333,238 (22%)	0.167	13,706 (15%)	13,247 (15%)	0.014
Polycystic kidney	35 (0%)	804 (0%)	0.006	35 (0%)	24 (0%)	0.007
Postural orthostatic tachycardia syndrome	98 (0%)	2082 (0%)	0.008	98 (0%)	67 (0%)	0.011
Pregnancy	4559 (5%)	30,869 (2%)	0.166	4484 (5%)	4946 (6%)	0.023
Sleep apnea	7559 (8%)	206,021 (13%)	0.163	7539 (8%)	7172 (8%)	0.015
Spondylosis	8578 (10%)	230,654 (15%)	0.169	8569 (10%)	7948 (9%)	0.024
Surgical procedures (spine)	179 (0%)	9547 (1%)	0.066	179 (0%)	175 (0%)	0.001
Surgical procedures (spine/cord)	1047 (1%)	32,043 (2%)	0.074	1043 (1%)	942 (1%)	0.011
Visit: emergency	15,629 (17%)	487,797 (32%)	0.342	15,602 (18%)	14,900 (17%)	0.021
Visit: inpatient encounter	6392 (7%)	476,545 (31%)	0.642	6392 (7%)	6373 (7%)	0.001
Visit: observation encounter	726 (1%)	56,994 (4%)	0.197	726 (1%)	728 (1%)	0.000

*Note*: After age/gender, variables are listed alphabetically. Counts <10 are rounded up to 10 for deidentification purposes.

Abbreviations: MRI, magnetic resonance imaging; NA, not applicable; PTE, physical therapist‐supervised therapeutic exercise; SMD, standardized mean difference; SMT, spinal manipulative therapy.

### Key results

**FIGURE 1:**
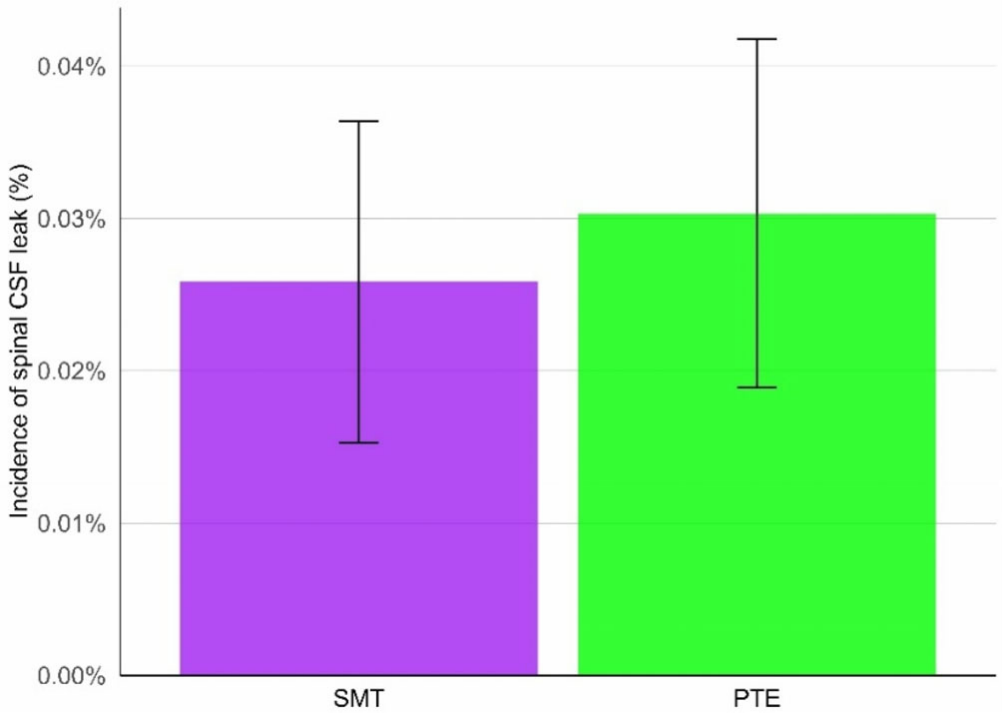
Incidences of spinal cerebrospinal fluid leak after propensity matching. Incidence in the spinal manipulative therapy cohort (purple) approximates that of the physical therapist‐supervised therapeutic exercises cohort (green), with substantial overlap in 95% confidence intervals. CSF, cerebrospinal fluid; PTE, physical therapist‐supervised therapeutic exercises; SMT, spinal manipulative therapy.

**FIGURE 2:**
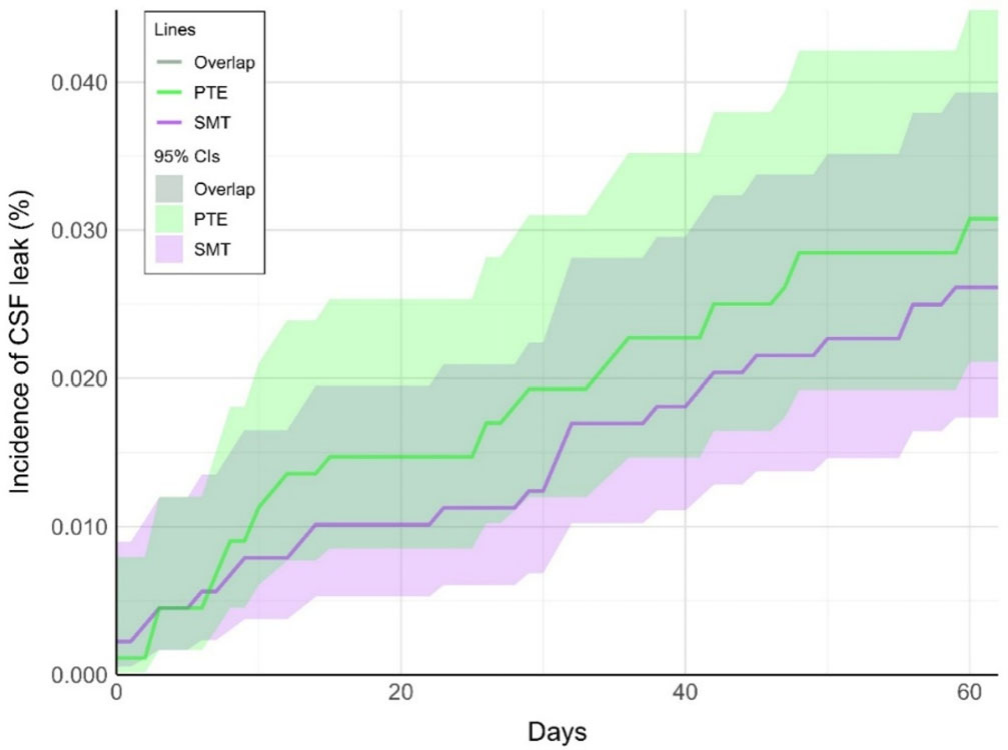
Cumulative incidences of spinal cerebrospinal fluid leak per cohort. The incidence for the spinal manipulative therapy cohort is shown in purple, and the incidence for the physical therapist‐supervised therapeutic exercise cohort is shown in green, over the 61‐day (2‐month) follow‐up. Shaded regions indicate 95% confidence intervals and overlapping incidences and confidence intervals are shown in darker green. CSF, cerebrospinal fluid; PTE, physical therapist‐supervised therapeutic exercises; SMT, spinal manipulative therapy.

After matching, there was no significant association between SMT and spinal CSF leak incidence or risk compared to PTE (both cohorts: 0.03% [95% CI: 0.02–0.04%]; RR = 0.85 [95% CI: 0.49–1.49]; *p* = .572). Adding SIH to the primary outcome yielded similar results, also having similar incidence and risk between cohorts (both cohorts: 0.03% [95% CI: 0.02%–0.04%]; RR = 0.82 95% CI: [0.47–1.43]; *p* = .484). Neither outcome demonstrated a significant risk difference between cohorts. Total and cumulative spinal CSF leak incidences are shown in Figures 1 and 2, respectively, and all key results are shown in Table 2.

**TABLE 2:** Key results.

	Before matching		After matching	
	SMT	PTE	SMT	PTE
Number of patients	89,753	1,527 758	89,042	89,042
Spinal CSF leak^a^				
*n*	23	446	23	27
% (95% CI)	0.03% (0.02% to 0.04%)	0.03% (0.03% to 0.03%)	0.03% (0.02% to 0.04%)	0.03% (0.02% to 0.04%)
*N* per 100,000 (95% CI)	25.6 (15.2 to 36.1)	29.2 (26.5 to 31.9)	25.8 (15.3 to 36.4)	30.3 (18.9 to 41.8)
RR (95% CI)	0.88 (0.58 to 1.33; *p* = .542)	Reference	0.85 (0.49 to 1.49; *p* = .572)	Reference
RD (95% CI)	−0.00% (−0.01% to 0.01%; *p* = .542)	Reference	−0.00% (−0.02% to 0.01%; *p* = .572)	Reference
Spinal CSF leak or spontaneous intracranial hypotension^b^				
*n*	23	478	23	28
% (95% CI)	0.03% (0.02% to 0.04%)	0.03% (0.03% to 0.03%)	0.03% (0.02% to 0.04%)	0.03% (0.02% to 0.04%)
*N* per 100,000 (95% CI)	25.6 (15.2 to 36.1)	31.3 (28.5 to 34.1)	25.8 (15.3 to 36.4)	31.4 (19.8 to 43.1)
RR (95% CI)	0.82 (0.54 to 1.24; *p* = .349)	Reference	0.82 (0.47 to 1.43; *p* = .484)	Reference
RD (95% CI)	−0.01% (−0.01% to 0.01%; *p* = .349)	Reference	−0.01% (−0.02% to 0.01%; *p* = .484)	Reference

Abbreviations: CI, confidence interval; CSF, cerebrospinal fluid; PTE, physical therapist‐supervised therapeutic exercise; RD, risk difference; RR, risk ratio; SMT, spinal manipulative therapy.

^a^ Primary outcome.

^b^ Secondary outcome.

### Data quality

**FIGURE 3:**
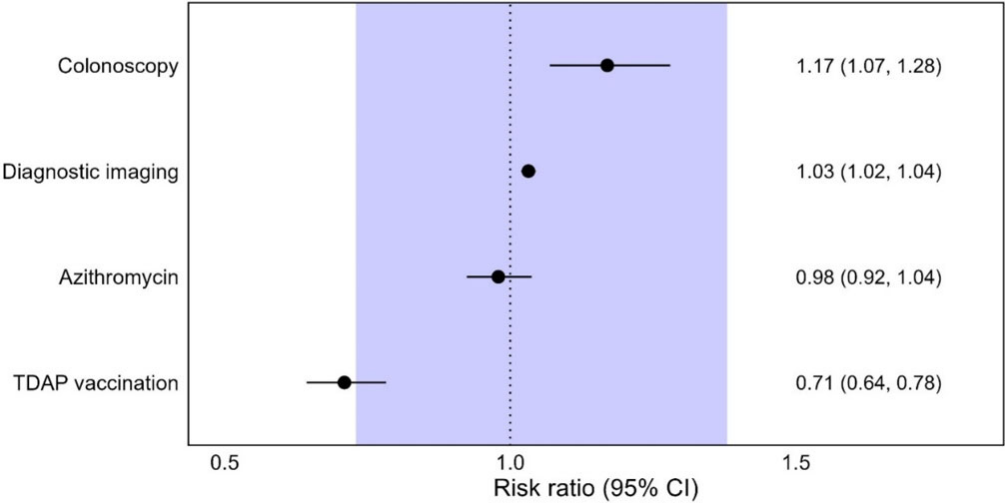
Negative control outcomes after matching. Point estimates and 95% confidence intervals (error bars) are shown for risk ratios for each. The blue area represents risk ratio estimates of 0.73 to 1.38, indicating the predetermined bounds for negative control outcome balance while the vertical dashed line indicates the null value of 1.0. TDAP, tetanus, diphtheria, pertussis.

Following matching, the two cohorts' propensity score densities overlapped substantially and all SMD values were optimally balanced (Supplementary File S1, Figures S2 and S3). The proportion of patients with an “unknown gender” was approximately 0% per cohort (SMD = 0.030) while all patients had known age. The mean follow‐up duration in days was (SD) 60.2 (5.9) in the SMT cohort and 59.9 (7.2) in the PTE cohort, which was not meaningfully different (SMD = 0.044). The median follow‐up duration for both cohorts was 61 days with an interquartile range of 0 days. The total duration of follow‐up in person‐years was 14,671 in the SMT cohort and 14,600 in the PTE cohort. An adequate proportion of patients had at least 61 days of follow‐up per cohort (SMT: 97.5%, PTE: 97.0%). Follow‐up metrics are visualized in Supplementary File S1, Figure S4. All but one negative control outcome fell within the predefined range approximating the null (Figure 3). Specifically, TDAP vaccination was slightly less common among those receiving SMT compared to PTE. However, since most negative control outcomes approximated the null, our predetermined criteria were met, further supporting adequacy of matching and cohort comparability. Together, these findings suggest no meaningful bias related to measured confounding variables, follow‐up duration, health care engagement, or ascertainment.

### Follow‐up care

In the SMT cohort, the mean follow‐up SMT visits (SD) was 5.1 (4.4) with a median of 4. Including the initial SMT visit, this translates to approximately 543,156 SMT visits across the cohort's 89,042 patients including the index date and follow‐up. In the SMT cohort, 34,603 patients received physical therapy‐related intervention(s) or reevaluation(s) whereas 71,415 patients in the PTE cohort received these therapies during the 61‐day follow‐up. Accordingly, the incidence and likelihood of receiving any physical therapy intervention or reevaluation during follow‐up was significantly lower in the SMT cohort compared to PTE cohort (38.9% vs. 80.2%; RR = 0.49 [95% CI: 0.48–0.49]; *p* < .001). Among individuals receiving physical therapy‐related follow‐up visits, the mean count of these visits was slightly lower in the SMT cohort [SD] (mean = 4.5 [4.1]; median = 3) versus PTE cohort (mean = 4.6 [3.8]; median = 4). Total and cumulative incidence plots for this outcome are shown in Figures S5 and S6.

## DISCUSSION

As the first adequately powered study on the topic to date with 178,084 total patients, the present study found no statistically significant or clinically meaningful association between receiving SMT and developing spinal CSF leak compared to matched controls receiving PTE without manual therapy. Additionally, our secondary outcome, which added SIH alongside the primary outcome of spinal CSF leak, led to similar findings of no statistically significant association. Our findings align with the general understanding that spinal CSF leaks may be spontaneous, with only a subset of cases linked to identifiable mechanical triggers.^1^, ^26^ Considering the incidence of CSF leaks was similar between the SMT and PTE cohorts, our results do not support the idea that SMT delivered by chiropractors uniquely contributes to development of CSF leaks.

The observed incidence of spinal CSF leak in the present study should be cautiously compared to that of previous studies due to differences in population demographics and study methods. However, the incidence of spinal CSF leak of 0.03% in both cohorts, which translates to 30 per  100,000 people per 3 months, was much higher than the reported incidence of this condition in the general population (ie, 5 per 100,000 person per 12 months^2^). Given this finding, it is possible that spinal CSF leak is more common among adults seeking care for spinal disorders in general. Although additional research is needed to corroborate the epidemiological characteristics of spinal CSF leak across populations with spinal pain, clinicians of any discipline who manage spinal pain should be aware of this condition as a potential etiology of symptoms.

Protopathic bias may explain the apparent discrepancy between our findings and previous case reports suggesting that SMT could trigger spinal CSF leak. In several case reports where SMT was suggested to trigger spinal CSF leak and/or SIH, patients had preceding symptoms such as neck pain.^35^, ^36^, ^50^, ^51^ Accordingly, these baseline symptoms could have mimicked more benign neuromusculoskeletal conditions while also being prodromal symptoms of undiagnosed CSF leaks. The similar incidence of spinal CSF leak in our active comparator, which did not receive SMT, suggests that underlying symptoms rather than SMT itself may have explained the previously reported cases of spinal CSF leaks occurring after SMT. In our study, neck pain and headache were common in both cohorts, affecting approximately 8%–9% and 14%–15% of patients per cohort for headache and neck pain, respectively. Given these are common symptoms of CSF leak,^3^ and common reasons patients seek SMT rendered by a chiropractor,^12^ it is plausible that previously reported cases reflect protopathic bias rather than a true causal relationship.

The proposed mechanism whereby SMT could lead to spinal CSF leak in the presence of spinal osteophytes is not supported by our findings.^7^ The present study used propensity matching to ensure the prevalences of spinal disc disorders and spondylosis were similar between cohorts at baseline. Given that spinal osteophytes are common among patients with spinal disc disorders and spondylosis, any potential association with CSF leak would not be expected to be uniquely related to SMT.

The present study findings support previous studies that suggested serious adverse events after SMT are rare. In general, the rate at which serious adverse events occur after SMT has been estimated at 1 per 2 million manipulations to 13 per 10,000 patients.^52^ In addition, one large observational study including 960,140 SMT sessions across 54,846 patients found two instances of serious adverse events, both of which were rib fractures, yet no instances of spinal CSF leak.^53^ We are unaware of any large cohort, chart review, or registry‐based study that has specifically aimed to estimate the incidence and risk of CSF leak after SMT other than the present study.

Our findings highlight that clinicians who manage spinal pain, such as chiropractors and physical therapists, should be aware of spinal CSF leaks as a potential cause of symptoms. Patients with a constellation of symptoms consistent with that of spinal CSF leak should receive further workup to rule out this condition, and clinicians may consider referring patients to neurology, neurosurgery, or emergency care if necessary.^54^ Although our results do not suggest a need to change SMT practice, clinicians using SMT should continue to apply judicious patient selection, appropriate SMT force modulation, clinical reasoning that includes weighing contraindications, precautions, alongside potential risk(s) and benefit(s) of SMT.^55^ Notably, our findings do not apply to patients with spinal infection, metastasis, or fracture, in whom SMT is already contraindicated.^55^

As a single study, these findings require additional corroboration. Future research could explore the potential association using other large datasets such as retrospective claims databases or prospective registries, which may offer adequate sample size. A case–control study could examine potential dose–response relationships between SMT and spinal CSF leak. Studies could also use additional active comparator groups not involving movement‐based treatments, such as primary care, outpatient spine specialist visits, or nonsteroidal anti‐inflammatory drug prescription. Modeling using finite element analysis could be used to simulate mechanical forces of SMT and help determine whether spinal osteophytes could exceed dural tolerance.^56^ Although we are unaware of any case reports of spinal CSF leaks following SMT in patients with recent epidural steroid injection, further research could explore whether this population may be at increased risk. Apart from SMT, broadly examining environmental or genetic predispositions (eg, connective tissue disorders) for spinal CSF leaks could help clarify factors that predispose subpopulations to this pathology.

Multiple licensed health professionals are trained in and administer SMT including chiropractors, physical therapists, osteopathic physicians, and others, with similar biomechanical characteristics in many instances regardless of the clinician administering the therapy.^5^, ^57^ Accordingly, our findings may plausibly generalize to SMT delivered by other clinicians. Despite the similarity of SMT across professions, we focused on chiropractor‐delivered SMT for pragmatic reasons of feasibility. Chiropractor‐specific CPT codes reliably identify SMT exposure in electronic health records^19^ and enabled an adequately powered analysis. In contrast, codes that might capture SMT performed by physical therapists or other clinicians are nonspecific (eg, CPT 97140: manual therapy) and include a heterogeneous array of manual therapies such as manual lymphatic drainage and manual traction targeting spinal and/or nonspinal regions, potentially mixing SMT with non‐SMT interventions. Additional interdisciplinary studies examining SMT across professions using other data sources will help establish whether our findings extend to SMT provided by nonchiropractic clinicians.

## STRENGTHS AND LIMITATIONS

Strengths of the study include its large sample size, multidisciplinary author team representing chiropractic, neurosurgery, and physical therapy, and the use of a propensity matching strategy to reduce confounding. However, several limitations should be acknowledged.

Considering our a priori sample size calculation included proportions and absolute risk difference, framing our primary analysis around the risk difference rather than the RR may have better aligned with an equivalence‐style interpretation. This strategy involves comparing confidence intervals with predefined equivalence regions rather than relying on statistical significance.^58^ Post hoc assessment using this framework leads to similar conclusions. After matching, the 95% CI for absolute risk difference in CSF leak was −0.02% to 0.01% (ie, −20 to +10 patients per 100,000), which excludes absolute differences of 0.05% (ie, 50 per 100,000), the margin used in our a priori sample‐size calculation. Accordingly, the observed data allow us, with 95% confidence, to rule out absolute differences of that magnitude. Another post hoc assessment based on RR point estimate magnitude corroborates our findings. Similar to our evaluation of negative control outcomes, the RR point estimate for CSF leak (0.85) lies within the nonmeaningful range of 0.73 to 1.38.^49^, ^59^ Our secondary expanded outcome of CSF leak or SIH demonstrated an almost identical risk difference with an identical 95% CI and a similarly nonmeaningful RR, further reinforcing these conclusions.

Calculation of our primary and secondary outcomes of RR, incidence proportions, and risk difference did not explicitly account for censoring. However, the cumulative incidence curves, which account for censoring, showed substantially overlapping 95% CIs throughout the follow‐up window, consistent with our primary analyses. Given the high completeness of follow‐up (≥97% with a median ≥ 61 days per cohort; ≤3% censored), any bias introduced by incomplete follow‐up is likely minimal, although some residual bias from differential censoring cannot be entirely excluded.

We did not impose a washout period or specific threshold for patients' duration of spinal disorder‐related symptoms prior to the index date nor were we able to evaluate symptom duration given the lack of data granularity. Although matching minimized between‐cohort differences in proxies of duration such as care use markers, some heterogeneity in symptom acuity/chronicity may remain.

Although there is overlap between the characteristics of patients seeking care from chiropractors versus physical therapists, potential differences in selection bias remain. Patients seeking physical therapist care may have slightly higher comorbidity burden, greater disability, and lower self‐rated health.^17^, ^23^, ^60^, ^61^ Our study design likely helped mitigate these differences as patients were drawn from large health care organizations and our matching strategy incorporated a broad set of demographics, comorbidities, health care use, spinal disorder‐related, and other variables. Additionally, given the short follow‐up window and the absence of evidence linking factors such as self‐rated health to CSF leak risk, we suspect any residual selection bias would be unlikely to substantially influence our findings.

We were unable to verify the specific type of spinal CSF leak (eg, dural tear or ruptured meningeal diverticula^29^) or verify its presence or location (eg, thoracic or lumbar and anterior or posterior) via imaging. There is also the potential for unmeasured confounding, particularly from undocumented genetic factors,^2^ or the severity of conditions like spondylosis or disc degeneration, which may increase the risk of spinal CSF leak and/or SIH.^62^ Outcome misclassification, such as false negatives or positives, remains possible due to the inherent challenges in CSF leak diagnosis.^34^ Additionally, we were unable to assess whether specific types of SMT were associated with greater risk, as the dataset does not describe the SMT technique(s) used. This could be relevant as forces applied to the spine may differ by technique. Lastly, the findings may have limited generalizability to countries outside the United States, where SMT, physical therapy practices, or evaluations for spinal CSF leak may differ. The present findings may not be generalizable to other forms of SMT not provided by a chiropractor,^9^ as spinal manipulative techniques may vary outside of this professional health discipline. Findings are also not generalizable to those with serious spinal pathology or recent spinal injection considering these patients were excluded.

## CONCLUSIONS

This large cohort study found no increased risk of spinal CSF leak following chiropractic SMT compared to PTE. However, as undiagnosed leaks may mimic musculoskeletal spinal issues with neck pain or headaches, clinicians should suspect CSF leak in atypical cases such as headaches having an orthostatic component and refer these patients for appropriate neurological, neurosurgical, or emergency care. Although limited to chiropractor‐delivered SMT, these findings may extend to SMT provided by other clinicians that should be examined in future interdisciplinary studies. Future research is also needed for corroboration potentially using alternative datasets or comparators, case–control or dose–response models, or simulated SMT.

## FUNDING INFORMATION

This project is supported by the Clinical and Translational Science Collaborative of Northern Ohio which is funded by the National Institutes of Health, National Center for Advancing Translational Sciences, Clinical and Translational Science Award grant, UM1TR004528. The study did not receive a specific grant and the funders did not play a role in the development of the study protocol or any key decision making. The work conducted by R.T. received support from the Elisabeth Severance Prentiss Foundation (Cleveland, OH) through general funding. The study did not receive a specific grant and the funders were not involved in developing the study protocol or any key decisions.

## ETHICS STATEMENT

The University Hospitals Institutional Review Board (IRB; Cleveland, OH, US) considers studies using deidentified data from the online TriNetX platform Not Human Subjects Research thereby exempting the present study from institutional review board review and waiving the need for consent.

## DISCLOSURE

Robert J. Trager acknowledges that he has received royalties as the author of two texts on the topic of sciatica. The other authors have declared no competing interests.

## Supporting information


**Data S1.** Supporting Information.

## Data Availability

Deidentified, aggregated data for baseline characteristics, the primary outcome, and data used to plot propensity score densities and cumulative incidences are available in a public figshare repository (https://doi.org/10.6084/m9.figshare.28464683).
