# Supplementary material for: No increased risk of spinal cerebrospinal fluid leak after spinal manipulative therapy: A retrospective cohort study
Source: PM R. 2025 Dec 26;18(8):874–84. doi: 10.1002/pmrj.70072 (PMC13098789; doi:10.1002/pmrj.70072)
Supplement: Supplementary file 1 [file PMRJ-18-874-s001.pdf]

Supplemental File 1

For: No increased risk of spinal cerebrospinal fluid leak after spinal manipulative therapy: a retrospective cohort study

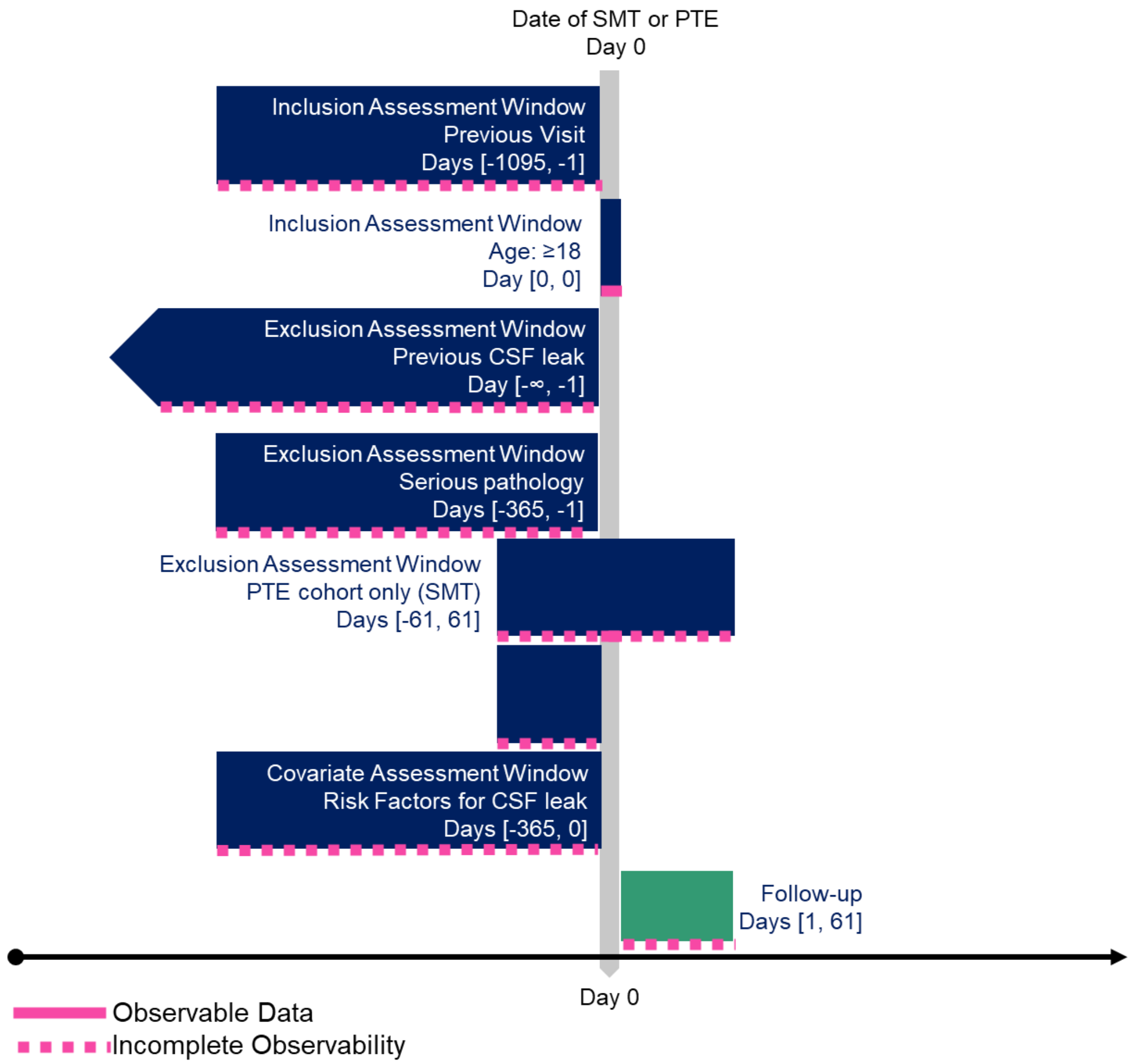

Figure S1: Visualization of study design. The gray vertical arrow shows the index date (day 0; cohort entry date), which is the first date patients meet eligibility criteria and receive chiropractic spinal manipulative therapy (SMT) or physical therapist-supervised therapeutic exercise (PTE). Text in blue boxes describes study eligibility criteria and their assessment during time windows in the specified number of days in relation to the index date ([#, #]). Cerebrospinal fluid (CSF) leak exclusion spans as long as preceding data are available per patient (-∞). Figure created using a template from Wang et al [1].

Table S1: Exclusion criteria. Abbreviations: any preceding duration of time available in the patient's data (-∞), cerebrospinal fluid (CSF); Current Procedural Terminology (CPT); International Classification of Diseases, 10th Edition (ICD-10); ICD-10 Procedure Coding System (ICD-10-PCS)

ICD-10 Codes	Definition	Window (days)
Exclusions for both cohorts		
A17, A18.01, G00-G09, M46.2, M46.3, M46.4, M46.5 (ICD-10)	Spinal infection: Tuberculosis of nervous system or spine; inflammatory diseases of the central nervous system (e.g., abscess); osteomyelitis of vertebra, infection of intervertebral disc (pyogenic), discitis, or other infective spondylopathies	-365 to -1
C41.2, C41.4, C70.1, C72.0, C72.1, C79.4, (ICD-10)	Spinal malignancy: Malignant neoplasm of vertebral column, pelvic bones, sacrum, and coccyx, spinal cord, spinal meninges, unspecified part of nervous system, or cauda equina	-365 to -1
M48.4, M48.5, M80.08, M80.88, S12, S22.0, S32.0 (ICD-10)	Spinal fracture: Fatigue fracture of vertebra; collapsed vertebra; age-related osteoporosis with current pathological fracture, vertebra(e) other osteoporosis with current pathological fracture, vertebra(e); fracture of cervical vertebra and other parts of neck; fracture of thoracic vertebra; fracture of lumbar vertebra	-365 to -1
00220 (CPT)	Anesthesia for intracranial procedures; CSF shunting procedures	-∞ to -1
1011079 (CPT)	CSF flow, imaging	-∞ to -1
1009323 (ICD-10)	CSF shunt procedures	-∞ to -1
10359005 (SNOMED)	Revision of obstructed valve in CSF shunt system	-∞ to -1
59916001 (SNOMED)	CSF flow imaging, shunt evaluation	-∞ to -1
Z98.2 (ICD-10)	Presence of CSF drainage device	-∞ to -1
G96.0 (ICD-10)	CSF leak	-∞ to -1
G97.0 (ICD-10)	CSF leak from spinal puncture	-∞ to -1
G97.2 (ICD-10)	Intracranial hypotension following ventricular shunting	-∞ to -1
G97.41 (ICD-10)	Accidental puncture or laceration of dura during a procedure	-∞ to -1
G97.83 (ICD-10)	Intracranial hypotension following lumbar CSF shunting	-∞ to -1
G97.84 (ICD-10)	Intracranial hypotension following other procedure	-∞ to -1
00U2 (ICD-10-PCS)	Supplement dura mater (dural graft)	-∞ to -1
1004038; 1009340 (CPT)	Surgical procedures on the spine (vertebral column); surgical procedures on the spine and spinal cord (includes injections, spinal cord stimulator procedures)	-61 to -1
G97.1 (ICD-10)	Headache due to lumbar puncture	-61 to -1
O74.5 (ICD-10)	Spinal and epidural anesthesia-induced headache during labor and delivery	-61 to -1
O89.4 (ICD-10)	Spinal and epidural anesthesia-induced headache during the puerperium	-61 to -1
R51.0 (ICD-10)	Headache with orthostatic component, not elsewhere classified	-61 to -1
009U; 3E0R, 3E0S (ICD-10-PCS)	Spinal canal (drainage); spinal canal or epidural space (introduction)	-61 to -1
18946005; 231249005; 277762005 (SNOMED)	Epidural anesthesia; spinal anesthesia; lumbar puncture	-61 to -1
96450 (CPT)	Chemotherapy administration, into CNS (eg, intrathecal), requiring and including spinal puncture	-61 to -1
J2274 (CPT)	Injection, morphine sulfate, preservative-free for epidural or intrathecal use, 10 mg	-61 to -1
Various	Outcome definition: Spinal CSF leak (manuscript Table 4)	-∞ to -1
Additional exclusions for the physical therapy-supervised exercise cohort		
98940, 98941, 98942 (CPT)	Chiropractic manipulative treatment, spinal	-61 to 61
1013558 (CPT)	Osteopathic manipulative treatment	-61 to 61
97140 (CPT)	Manual therapy	-61 to 61
97124 (CPT); 8E0KX1Z (ICD-10-PCS); 387854002 (SNOMED)	Massage; therapeutic massage; massage physiotherapy	-61 to 61

Table S2: Variables controlled for in propensity score matching. Abbreviations: International Classification of Diseases, 10th Edition (ICD-10); custom TriNetX code (*)

Variable	Description	Reference
**Demographics**	Patient age, sex	
**Comorbidities**		
E66 (ICD-10)	Overweight and obesity	[2]
F40.240 (ICD-10)	Claustrophobia	[3]
G43 (ICD-10)	Migraine	[4]
G47.3 (ICD-10)	Sleep apnea	[5]
G90.A (ICD-10)	Postural orthostatic tachycardia syndrome (POTS)	[6,7]
G93.2 (ICD-10)	Benign intracranial hypertension	[8]
G96 (ICD-10)	Other disorders of central nervous system (includes perineural cysts, meningeal disorders)	[9]
I95.1 (ICD-10)	Orthostatic hypotension	[6,7]
M35.7 (ICD-10)	Hypermobility syndrome	[7]
M45 (ICD-10)	Ankylosing spondylitis	[10]
M47 (ICD-10)	Spondylosis	[11]
M50 (ICD-10)	Cervical disc disorders	[9]
M51 (ICD-10)	Thoracic, thoracolumbar, and lumbosacral disc disorders	[9]
M54.2 (ICD-10)	Neck pain	[12]
O00-O9A (ICD-10)	Pregnancy, childbirth and the puerperium	[13]
Q07.0 (ICD-10)	Arnold-Chiari syndrome	[4,7]
Q61.2 (ICD-10)	Autosomal dominant polycystic kidney disease	[14]
Q79.6 (ICD-10)	Ehlers-Danlos syndromes	[7,11]
Q85.01 (ICD-10)	Neurofibromatosis, type I	[14]
Q87.4 (ICD-10)	Marfan syndrome	[7,11]
Q87.89 (ICD-10)	Congenital malformation syndromes, not elsewhere classified (includes Loeys-Dietz syndrome)	[7]
R51 (ICD-10)	Headache	[12]
V00-Y99 (ICD-10)	External causes of morbidity (trauma)	[15]
**Procedures**		
1007385 (CPT)	Bariatric surgery procedures	[16]
1004038 (CPT)	Surgical procedures on the spine (vertebral column)	[17]
1009340 (CPT)	Surgical procedures on the spine and spinal cord (includes injections and spinal cord stimulator procedures)	[17]
**Healthcare utilization and socioeconomic markers**		
70551 (CPT)	Magnetic resonance (eg, proton) imaging, brain	NA
1010367 (CPT)	Diagnostic radiology procedures of the spine and pelvis	NA
1010399 (CPT)	Magnetic resonance (eg, proton) imaging, cervical	NA
1010402 (CPT)	Magnetic resonance (eg, proton) imaging, thoracic	NA
1010405 (CPT)	Magnetic resonance (eg, proton) imaging, lumbar	NA
5956 (RxNorm)	Iohexol (contrast agent used in myelography)	NA
A9579 (HCPCS)	Injection, gadolinium-based magnetic resonance contrast agent, not otherwise specified (nos), per ml	NA
EMER*	Visit: Emergency	NA
IMP*	Visit: Inpatient	NA
OBSENC*	Visit: Observation	NA
Z55-Z65 (ICD-10)	Adverse socioeconomic and psychosocial circumstances (i.e., social determinants of health) [18,19]	NA

Table S3: Codes used to define spinal cerebrospinal fluid leak. Abbreviations: International Classification of Diseases, 10th Edition (ICD-10)

Variable	Description	Reference(s)
**Spinal CSF leak**		
03.95 (ICD-9-CM)	Spinal blood patch	[20,21]
3E0S3GC (ICD-10-PCS)	Epidural blood patch	[20]
447366001 (SNOMED)	Injection of autologous whole blood into spinal epidural space	[22]
62273 (CPT)	Injection, epidural, of blood or clot patch	[23]
63707 (CPT)	Repair of dural/cerebrospinal fluid leak, not requiring laminectomy	[24,25]
63709 (CPT)	Repair of dural/cerebrospinal fluid leak or pseudomeningocele, with laminectomy	[24,25]
63710 (CPT)	Dural graft, spinal	[25]
G96.02 (ICD-10)	Spinal cerebrospinal fluid leak, spontaneous	[26]
G96.09 (ICD-10)	Other spinal cerebrospinal fluid leak	[26]
G96.11 (ICD-10)	Dural tear	[26]

Table S4: Codes used to define spontaneous intracranial hypotension. Abbreviations: International Classification of Diseases, 10th Edition (ICD-10), Logical Observation Identifier Names and Codes (LOINC)

Variable	Description	Reference(s)
**Spontaneous intracranial hypotension**		
G96.81 (ICD-10)	Intracranial hypotension	[26]
60956-0 (LOINC)	Intracranial pressure (ICP); Filter: ≤ 4 mm Hg	[4,10]

Table S5: Codes used to identify physical therapy follow-up visits. Abbreviations: Current Procedural Terminology (CPT); International Classification of Diseases, 10th Revision Procedure Coding System (ICD-10-PCS); Systematized Nomenclature of Medicine (SNOMED)

Code	Definition
1013490 (CPT)	Physical medicine and rehabilitation modalities, including supervised physical medicine and rehabilitation modalities (i.e., CPT codes 97010, 97012, 97014, 97016, 97018, 97022, 97024, 97026, 97028), constant attendance modalities (i.e., 97032, 97033, 97034, 97035, 97036, 97037), unlisted modalities (i.e., 97037)
1013510 (CPT)	Physical medicine and rehabilitation therapeutic procedures, including therapeutic procedures (i.e., CPT codes 97110, 97112, 97113, 97116, 97124), work hardening/conditioning, therapeutic interventions that focus on cognitive function (e.g., attention, memory, reasoning, executive function, problem solving, and/or pragmatic functioning) and compensatory strategies to manage the performance of an activity (e.g., managing time or schedules, initiating, organizing, and sequencing tasks), direct (one-on-one) patient contact, and caregiver training
91251008 (SNOMED)	Physical therapy procedure
F07 (ICD-10-PCS)	Motor treatment (e.g., therapeutic exercise)
G0283 (HCPCS)	Electrical stimulation (unattended), to one or more areas for indication(s) other than wound care, as part of a therapy plan of care
398074008 (SNOMED)	Thermotherapy with hot packs
20560 (CPT)	Needle insertion(s) without injection(s); 1 or 2 muscle(s)
20561 (CPT)	Needle insertion(s) without injection(s); 3 or more muscles
97799 (CPT)	Unlisted physical medicine/rehabilitation service or procedure: This code is appended when no other specific CPT code is applicable, and may represent hydro massage, forms of bioelectrical or electromagnetic stimulation, hands-free ultrasound, or other unlisted physical medicine or rehabilitation therapies [27].
97002 (CPT)	Physical therapy re-evaluation (deprecated 2018)
97164 (CPT)	Re-evaluation of physical therapy established plan of care, requiring these components: An examination including a review of history and use of standardized tests and measures is required; and Revised plan of care using a standardized patient assessment instrument and/or measurable assessment of functional outcome Typically, 20 minutes are spent face-to-face with the patient and/or family

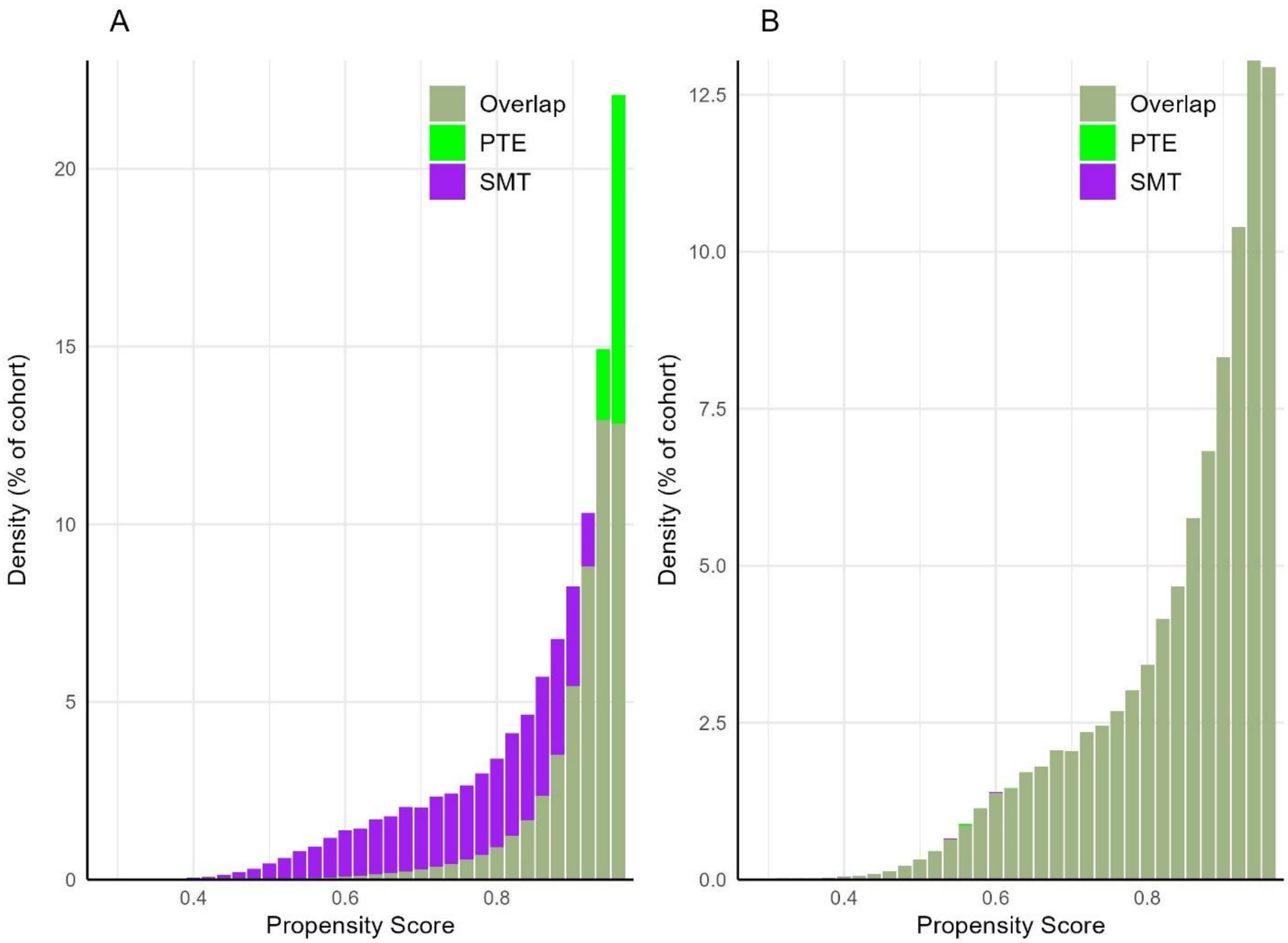

Figure S2: Propensity score densities. Density scores before (A) and after (B) matching. Purple bars represent the spinal manipulative therapy (SMT) cohort while green bars represent the PTE cohort. Following matching, densities are superimposed (dark green) indicating adequate balance of covariates.

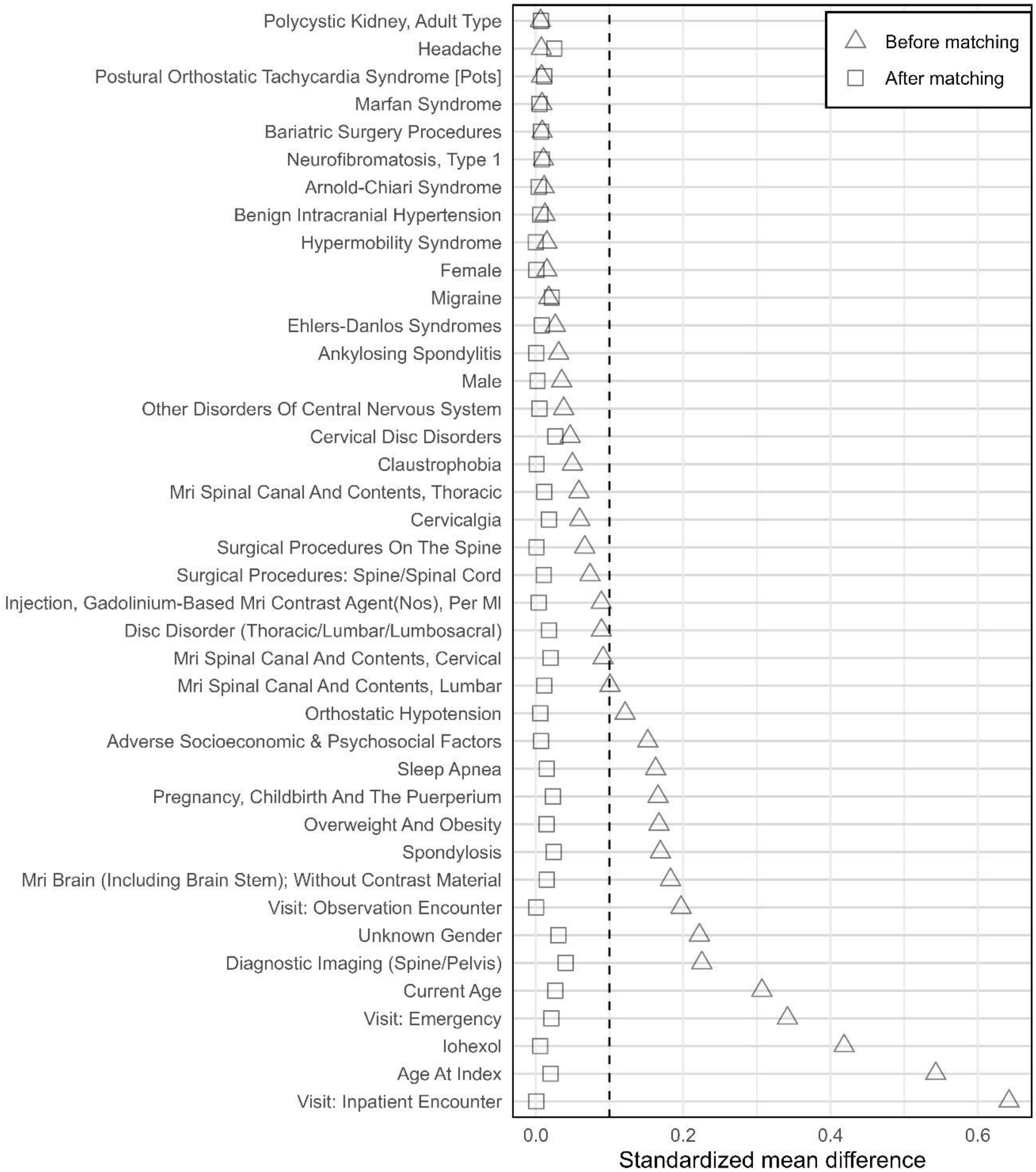

Figure S3: Covariate balance plot (Love plot) showing standardized mean differences (SMDs) before and after matching. These represent between-cohort balance of matched covariates. The vertical dashed line at SMD of 0.1 indicates the threshold for optimal balance [28,29]. Triangles show before-matching SMDs, while squares show after-matching SMDs. The plot illustrates optimal covariate balance across all variables following matching. Plot created using R and R studio (version 4.2.2, Vienna, AT [30]) and the ggplot2 package [31].

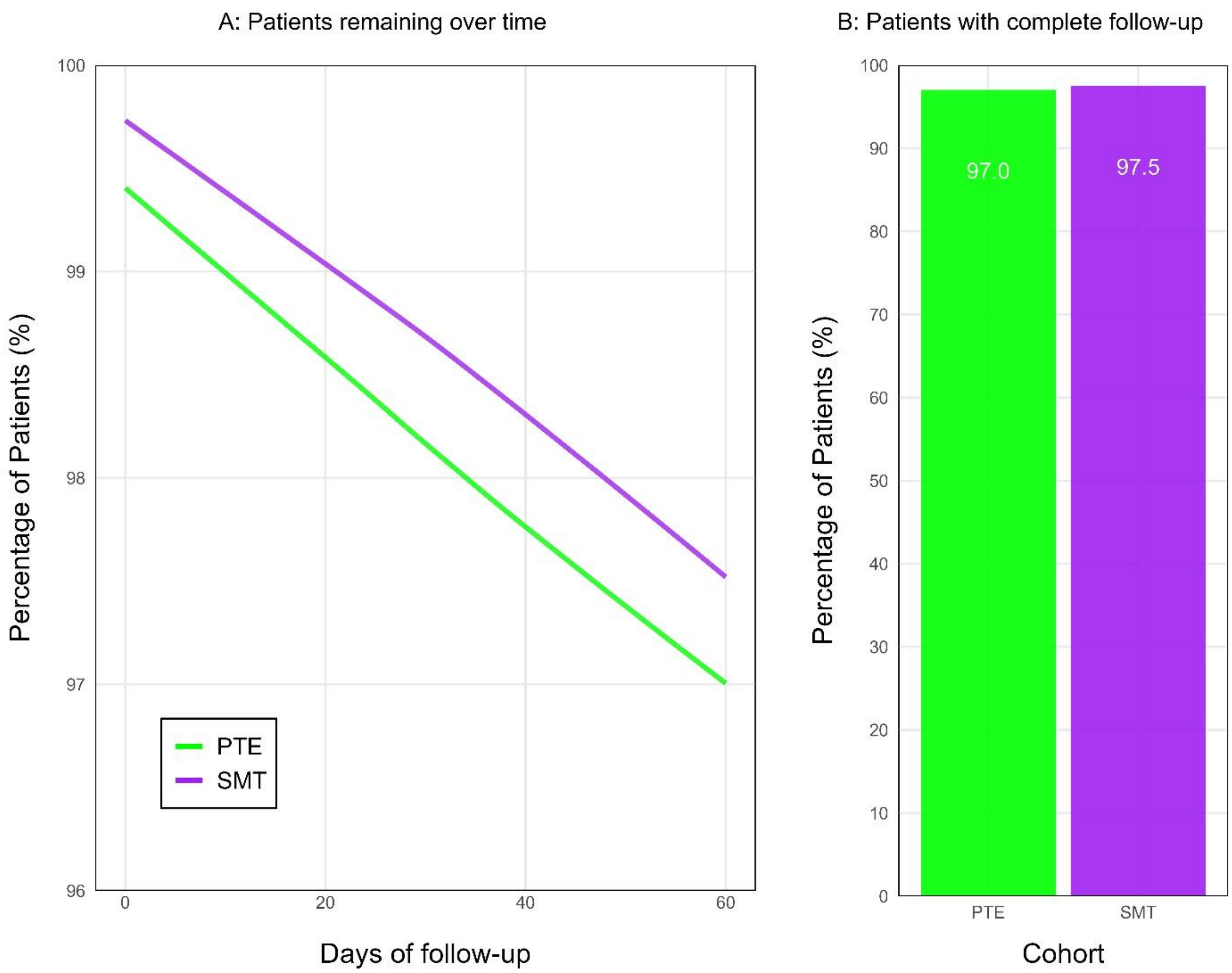

Figure S4: Follow-up data. A: This graph shows the proportion of patients remaining per cohort per follow-up day, indicating the spinal manipulative therapy (SMT) cohort in purple and physical therapist-supervised therapeutic exercises (PTE) cohort in green, using locally estimated scatterplot smoothing. B: This bar graph shows the proportion of patients per cohort who had at least 61 days of follow-up available (i.e., SMT: 97.5%; PTE: 97.0%). Plots were made using R and R studio (version 4.2.2, Vienna, AT [30]) and the ggplot2 package [31].

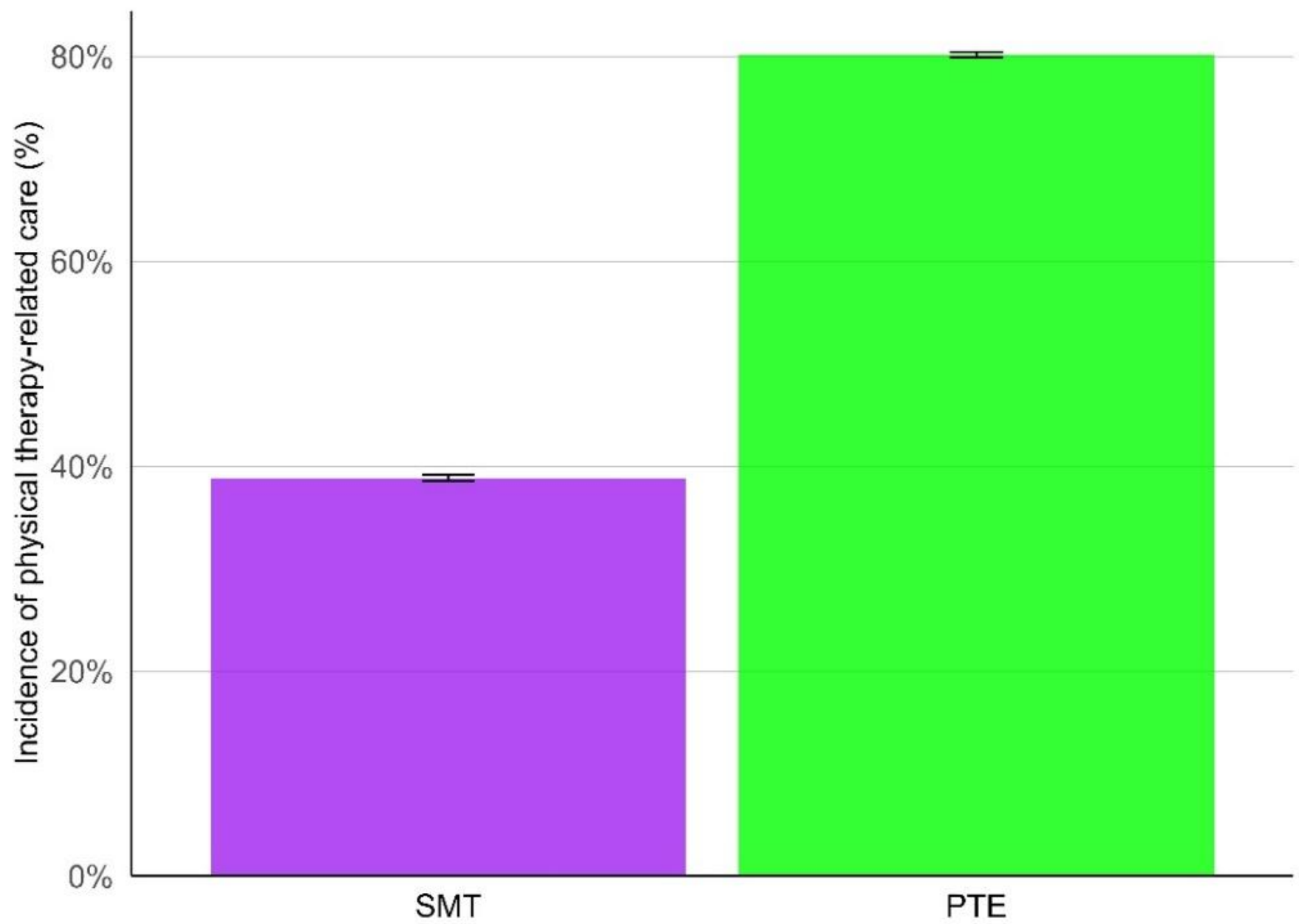

Figure S5: Total incidences of physical therapy-related care (i.e., procedures or re-evaluations) after propensity matching. Incidence in the spinal manipulative therapy (SMT) cohort (purple) is substantially lower than that of the physical therapist-supervised therapeutic exercises (PTE) cohort (green), with no overlap in 95% confidence intervals.

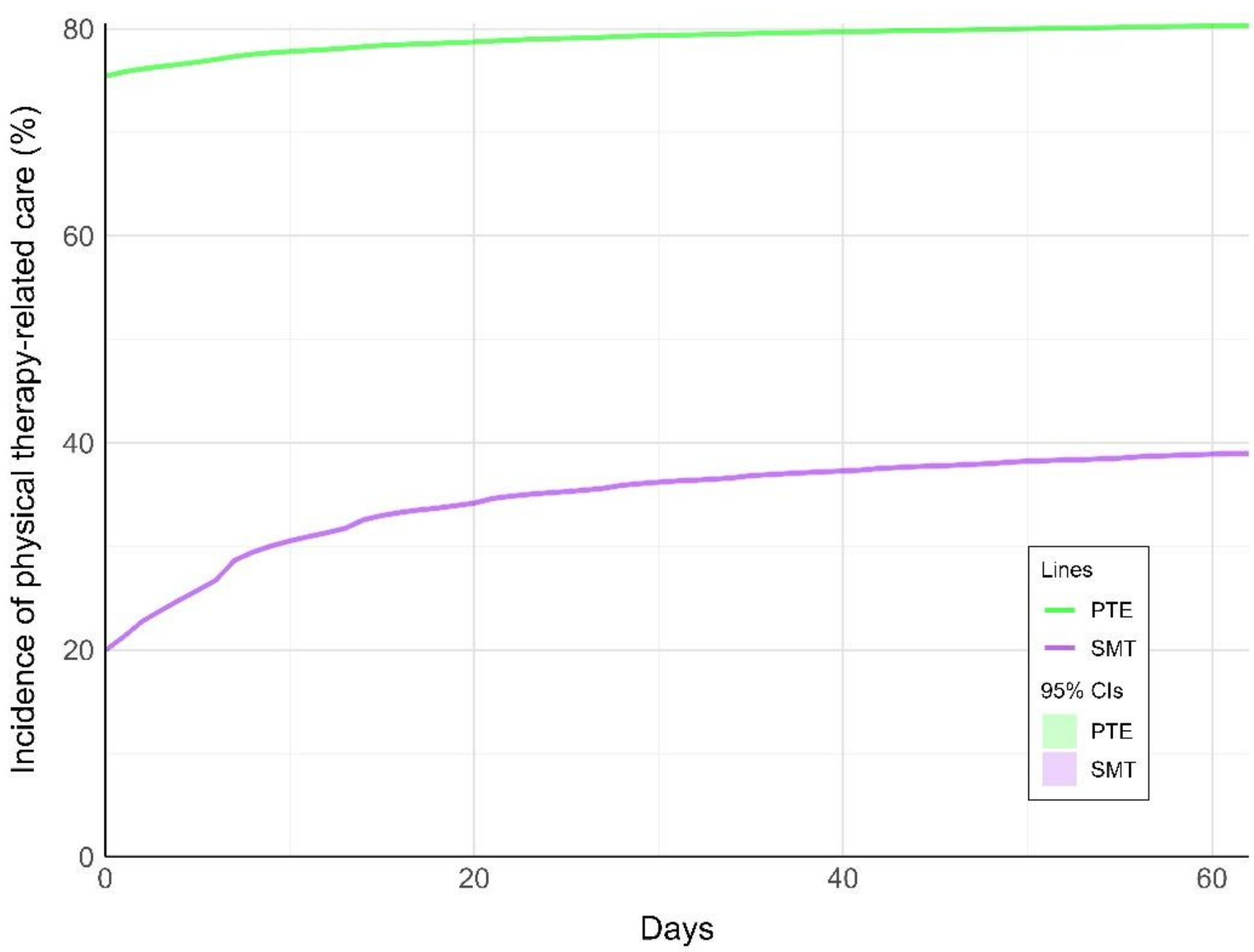

Figure S6: Cumulative incidences of physical therapy-related care (i.e., procedures or re-evaluations) per cohort after propensity matching. The incidence for the spinal manipulative therapy (SMT) cohort is shown in purple, while the incidence for the physical therapist-supervised therapeutic exercise (PTE) cohort is shown in green, over the 61-day (two-month) follow-up. Shaded regions indicate 95% confidence intervals while overlapping incidences and confidence intervals are shown in darker green.

References

1. Wang SV, Schneeweiss S. A Framework for Visualizing Study Designs and Data Observability in Electronic Health Record Data. Clin Epidemiol. 2022 Apr 29;14:601–8.

2. Mamlouk MD, Shen PY, Jun P, Sedrak MF. Spontaneous Spinal CSF Leaks Stratified by Age, Body Mass Index, and Spinal Level. AJNR Am J Neuroradiol. 2022 July;43(7):1068–72.

3. Martineau P, Chakraborty S, Faiz K, Shankar J. Imaging of the Spontaneous Low Cerebrospinal Fluid Pressure Headache: A Review. Can Assoc Radiol J. 2020 May 1;71(2):174–85.

4. Dobrocky T, Nicholson P, Häni L, Mordasini P, Krings T, Brinjikji W, Cutsforth-Gregory JK, Schär R, Schankin C, Gralla J, Pereira VM, Raabe A, Farb R, Beck J, Piechowiak EI. Spontaneous intracranial hypotension: searching for the CSF leak. Lancet Neurol. 2022 Apr;21(4):369–80.

5. Bakhsheshian J, Hwang MS, Friedman M. Association Between Obstructive Sleep Apnea and Spontaneous Cerebrospinal Fluid Leaks: A Systematic Review and Meta-analysis. JAMA Otolaryngol Neck Surg. 2015 Aug 1;141(8):733–8.

6. Kato Y, Hayashi T, Arai N, Tanahashi N, Takahashi K, Takao M. Spontaneous Intracranial Hypotension Associated with Postural Tachycardia Syndrome. Intern Med. 2019 May 22;58(17):2569.

7. Reinstein E, Pariani M, Bannykh S, Rimoin DL, Schievink WI. Connective tissue spectrum abnormalities associated with spontaneous cerebrospinal fluid leaks: a prospective study. Eur J Hum Genet. 2013 Apr;21(4):386–90.

8. Bidot S, Levy JM, Saindane AM, Oyesiku NM, Newman NJ, Biousse V. Do Most Patients With a Spontaneous Cerebrospinal Fluid Leak Have Idiopathic Intracranial Hypertension? J Neuro-Ophthalmol Off J North Am Neuro-Ophthalmol Soc. 2019 Dec;39(4):487–95.

9. Beck J, Ulrich CT, Fung C, Fichtner J, Seidel K, Fiechter M, Hsieh K, Murek M, Bervini D, Meier N, Mono ML, Mordasini P, Hewer E, Z'Graggen WJ, Gralla J, Raabe A. Diskogenic microspurs as a major cause of intractable spontaneous intracranial hypotension. Neurology. 2016 Sept 20;87(12):1220–6.

10. Schievink WI. Spontaneous Intracranial Hypotension. N Engl J Med. 2021 Dec 1;385(23):2173–8.

11. Jones MR, Shlobin NA, Dahdaleh NS. Spontaneous Spinal Cerebrospinal Fluid Leak: Review and Management Algorithm. World Neurosurg. 2021 June 1;150:133–9.

12. D'Antona L, Merchan MAJ, Vassiliou A, Watkins LD, Davagnanam I, Toma AK, Matharu MS. Clinical Presentation, Investigation Findings, and Treatment Outcomes of Spontaneous Intracranial Hypotension Syndrome: A Systematic Review and Meta-analysis. JAMA Neurol. 2021 Jan 4;78(3):1.

13. Rohatgi VK, Robbins MS, Roytman M, Chazen JL. Spontaneous Intracranial Hypotension in Pregnancy. Curr Pain Headache Rep. 2023 Nov 1;27(11):685–93.

14. Sung J, Lin M, Giantini-Larsen A, Kim A, Edasery D, Roytman M, Strauss S, Schweitzer AD, Park JK, Salama G. Cerebrospinal Fluid Leaks: Challenges in Localizing Spontaneous Spinal Leak Sites and Minimally Invasive Treatment. World Neurosurg. 2024 July 1;187:294–303.

15. Schievink WI, Louy C. Precipitating factors of spontaneous spinal CSF leaks and intracranial hypotension. Neurology. 2007 Aug 14;69(7):700–2.

16. Schievink WI, Goseland A, Cunneen S. Bariatric surgery as a possible risk factor for spontaneous intracranial hypotension. Neurology. 2014 Nov 11;83(20):1819–22.

17. Khazim R, Dannawi Z, Spacey K, Khazim M, Lennon S, Reda A, Zaidan A. Incidence and treatment of delayed symptoms of CSF leak following lumbar spinal surgery. Eur Spine J Off Publ Eur Spine Soc Eur Spinal Deform Soc Eur Sect Cerv Spine Res Soc. 2015 Sept;24(9):2069–76.

18. Heidari E, Zalmai R, Richards K, Sakthisivabalan L, Brown C. Z-code documentation to identify social determinants of health among Medicaid beneficiaries. Res Soc Adm Pharm RSAP. 2023 Jan;19(1):180–3.

19. Schulte A, Donovan AL, McAdam-Marx C. From access to equity: The devil is in “Z” details. J Manag Care Spec Pharm. 2022 June;28(6):685–7.

20. Lee A, Guglielminotti J, Janvier AS, Li G, Landau R. Racial and Ethnic Disparities in the Management of Postdural Puncture Headache With Epidural Blood Patch for Obstetric Patients in New York State. JAMA Netw Open. 2022 Apr 21;5(4):e228520.

21. Guglielminotti J, Landau R, Ing C, Li G. Temporal trends in the incidence of post-dural puncture headache following labor neuraxial analgesia in the United States, 2006–2015. Int J Obstet Anesth. 2021 Feb;45:90–8.

22. Medical Dictionary for Regulatory Activities Terminology (MedDRA) - Epidural blood patch - Classes | NCBO BioPortal [Internet]. [cited 2024 Sept 16]. Available from: https://bioportal.bioontology.org/ontologies/MEDDRA?p=classes&conceptid=10055034#details

23. Manchikanti L. Role of correct coding for interventional techniques. Pain Physician. 2001 Oct;4(4):381–99.

24. Murphy ME, Kerezoudis P, Alvi MA, McCutcheon BA, Maloney PR, Rinaldo L, Shepherd D, Ubl DS, Krauss WE, Habermann EB, Bydon M. Risk factors for dural tears: a study of elective spine surgery*. Neurol Res. 2017 Feb 1;39(2):97–106.

25. Arshi A, Wang C, Park HY, Blumstein GW, Buser Z, Wang JC, Shamie AN, Park DY. Ambulatory anterior cervical discectomy and fusion is associated with a higher risk of revision surgery and perioperative complications: an analysis of a large nationwide database. Spine J Off J North Am Spine Soc. 2018 July;18(7):1180–7.

26. Diagnostic coding - New diagnostic codes [Internet]. Spinal CSF Leak Foundation. 2020 [cited 2024 Sept 16]. Available from: https://spinalcsfleak.org/diagnostic-coding/

27. Highmark Medical Policy Bulletin Therapy Y-1 Physical Medicine [Internet]. 2008 [cited 2025 Sept 7]. Available from: https://secure.highmark.com/ldap/medicalpolicy/wpa-highmark/printerfriendly/Y-1-031.html

28. Austin PC. Balance diagnostics for comparing the distribution of baseline covariates between treatment groups in propensity-score matched samples. Stat Med. 2009;28(25):3083–107.

29. Stuart EA, Lee BK, Leacy FP. Prognostic score–based balance measures can be a useful diagnostic for propensity score methods in comparative effectiveness research. J Clin Epidemiol. 2013 Aug 1;66(8, Supplement):S84-S90.e1.

30. R Core Team. R: A Language and Environment for Statistical Computing [Internet]. Vienna, Austria: R Foundation for Statistical Computing; 2022. Available from: https://www.R-project.org/

31. Wickham H. ggplot2: Elegant Graphics for Data Analysis [Internet]. Springer-Verlag New York; 2016. Available from: https://ggplot2.tidyverse.org
